# Exploring the therapeutic potential and *in vitro* validation of baicalin for the treatment of triple-negative breast cancer

**DOI:** 10.3389/fphar.2025.1530056

**Published:** 2025-04-28

**Authors:** Yuan Ma, Ying Pan, Qiancheng Zhao, Chongheng Zhang, Haitao He, Lihua Pan, Jianling Jia, Aiping Shi, Yiming Yang, Wenfeng Zhang

**Affiliations:** ^1^ School of Basic Medicine, Changchun University of Traditional Chinese Medicine, Changchun, Jilin, China; ^2^ Department of Histology and Embryology, College of Basic Medical Sciences, Jilin University, Changchun, Jilin, China; ^3^ Department of Cell Biology and Medical Genetics, College of Basic Medical Sciences, Jilin University, Changchun, Jilin, China; ^4^ College of Basic Medical Sciences, Jilin University, Changchun, Jilin, China; ^5^ Department of Breast Surgery, General Surgery Center, The First Hospital of Jilin University, Changchun, China

**Keywords:** baicalin, triple-negative breast cancer, molecular dynamics simulation, molecular docking, network pharmacology

## Abstract

**Objective:**

To explore the mechanism of action of baicalin (BA) in the treatment of triple-negative breast cancer (TNBC) based on network pharmacology, molecular docking and molecular dynamics simulations and *in vitro* validation.

**Methods:**

The inhibitory effects of different concentrations of baicalin on the proliferation of MDA-MB-231, 4T1, MCF-7, and MCF-10A cell lines were evaluated by CCK8 assay with clone formation assay. Three compound target prediction platforms, Swiss Target Prediction, SEA and Pharmmapper, were used to predict baicalin-related targets, and mapped with the triple-negative breast cancer-related targets retrieved from GeneCards and OMMI databases to obtain the potential targets of baicalin for the treatment of triple-negative breast cancer; the STRING database and the STRING database and Cytoscape software were used to construct the protein interaction network and screen the core targets; GO and KEGG enrichment analyses were performed on the core targets; the binding of baicalin to the key targets of triple-negative breast cancer was verified by molecular docking and molecular dynamics simulation; and the expression of the relevant proteins was verified.

**Results:**

Baicalin showed more obvious antiproliferative effects on triple-negative breast cancer cell lines at certain concentrations, and had less effect on the proliferation of normal breast cells. A total of nine core targets of baicalin in the treatment of triple-negative breast cancer, including AKT1, ESR1, TNF-α, SRC, EGFR, MMP9, JAK2, PPARG, and GSK3B, were identified through the construction of the PPI protein interactions network and the ‘Traditional Chinese Medicine-Component-Target-Disease’ network, and a total of 252 targets related to the intersected targets were identified in the GO analysis. GO analysis enriched a total of 2,526 Biological process, 105 Cellular component and 250 Molecular function related to the intersecting targets; KEGG analysis enriched a total of 128 signaling pathways related to the intersecting targets; molecular docking results and molecular dynamics studies found that baicalin was able to interact with MMP9, TNF-α, JAK2, PPARG, GSK3B, and other core targets of baicalin for the treatment of triple-negative breast, MMP9, TNF-α, and JAK2 target proteins, and had significant changes in the expression levels of the target proteins.

**Conclusion:**

Baicalin inhibits the protein expression of MMP9, TNF-α and JAK2 and their related signaling pathways in the treatment of triple-negative breast cancer.

## 1 Introduction

Breast cancer (BC) has the highest incidence rate among all forms of cancer globally. In China, breast cancer stands as the foremost form of malignant tumor among women, and its incidence rate has been consistently on the rise in recent years, with triple-negative BC (TNBC) being a particularly aggressive and heterogeneous subtype (Siegel et al., 2024; Ji et al., 2024; Bray et al., 2024). The lack of clinically targetable genetic mutations in TNBC limits the use of targeted therapies (Thakur and Kutty, 2019). However, traditional Chinese medicines, which contain multiple active ingredients, often show therapeutic effects across various targets and pathways (Yang et al., 2021). This multi-targeted approach can offer a broader therapeutic strategy for clinical diseases like TNBC, where specific targeted therapies are lacking.

The Chinese medicinal herb scutellaria baicalensis has a long history of usage and documentation in the treatment of breast disorders (Zhao et al., 2016). Baicalin (BA), a flavonoid compound derived from the desiccated root of Scutellaria baicalensis (Bochoráková et al., 2003). has antitumor properties in addition to its conventional pharmacological activity, including antibacterial, heat-expelling, choleretic, and hepatoprotective actions (Li et al., 2020; Singh et al., 2021; Jiang et al., 2020). As one of the primary active components of Scutellaria baicalensis, BA has gained increased attention from academics and clinicians in recent years as an essential research direction to enhance Scutellaria baicalensis bioavailability and effectiveness. Literature indicates that BA suppresses PD-L1 expression and modulates the AKT/mTOR signaling pathway, potentially mitigating tumor immune tolerance *in vivo* and offering a novel approach for anti-tumor immunotherapy (Liu et al., 2024). Phase I clinical trials have demonstrated that oral administration of BA is safe for humans (Hayate and Shreesh, 2023). BA decreases serum malondialdehyde (MDA) levels, thereby reducing oxidative stress damage, and inhibits fatty acid synthase (FAS) activity, hindering tumor energy metabolism (Felice et al., 2022). In mouse xenograft models, BA (administered at 100 mg/kg via intraperitoneal injection) notably reduced the tumor volumes of MCF-7 and 4T1 (by 50%–60%) and exhibited synergistic effects when combined with doxorubicin. Immunohistochemistry revealed significant changes in the expression of p-AKT, LC3, and Bax in the BA-treated group (Yan et al., 2018). In radiation/chemotherapy-resistant MDA-MB-231/IR cells, BA restores sensitivity by upregulating the IFIT2 gene (negatively correlated with metastasis), decreasing the IC_50_ by 30% compared to parental cells (Koh et al., 2019).

**FIGURE 1:**
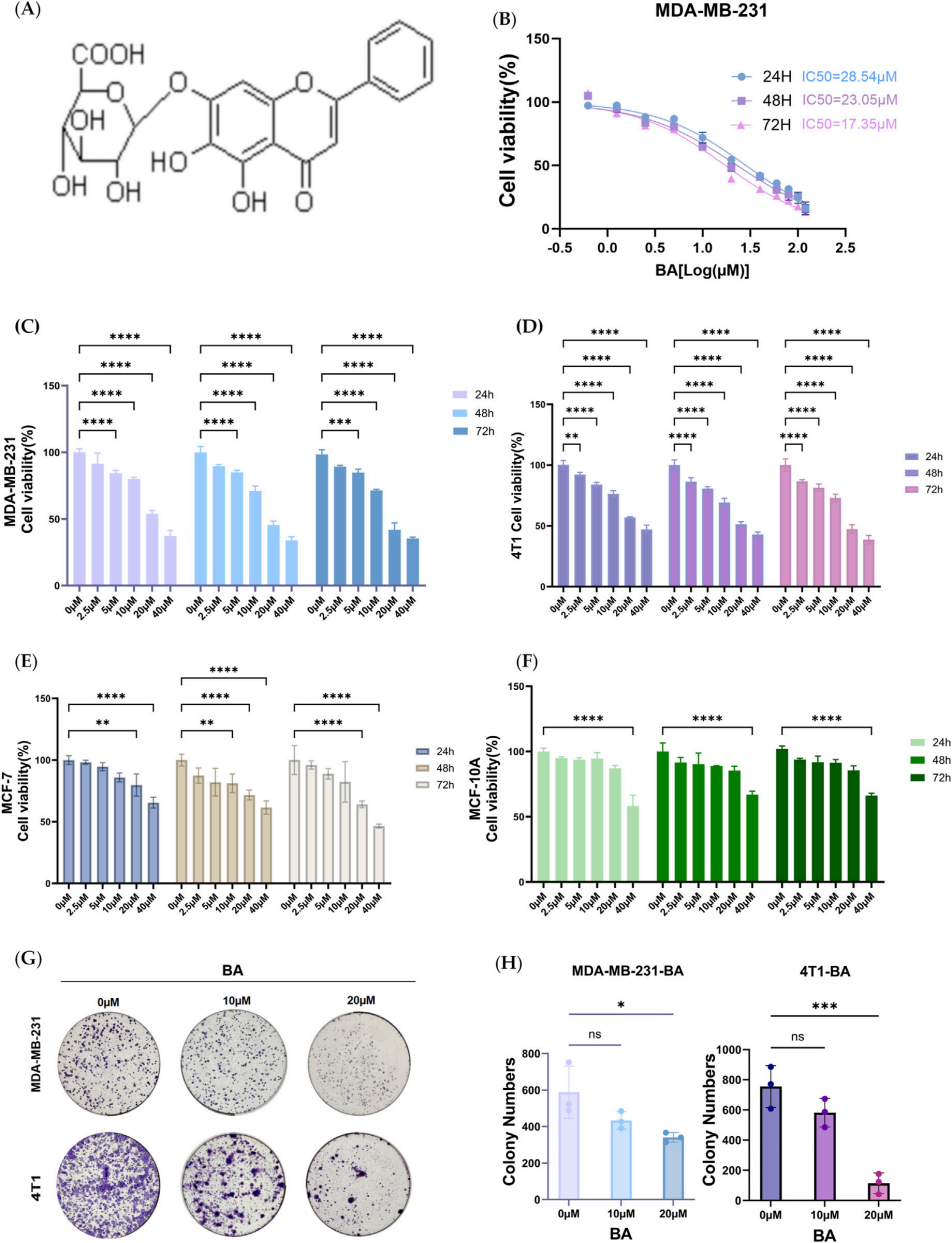
Inhibition of viability and proliferation of BC cells by BA. Figure shows **(A)** Chemical structure of BA **(B)** Half maximal inhibitory concentration of BA; **(C–F)** MDA-MB-231, 4T1, MCF-7, and MCF-10A cells treated with BA at concentrations of 0, 2.5, 5, 10, 20, and 40 µM for 24, 48, and 72 h; **(G–H)** The proliferation of MDA-MB-231 and 4T1 cells after treatment with BA at concentrations of 0, 10, and 20 μM. *p<0.05; **p<0.01; ***p<0.001.

Figure 1A depicts the molecular structure of BA, which includes Baicalein and a glucose molecule. This means that baicalein is an important component of baicalin. Baicalein is a flavonoid with a core structure based on a 1,4-naphthoquinone system, featuring multiple phenolic hydroxyl and ketone groups. These functional groups are crucial to the biological activity of baicalein. The phenolic hydroxyl groups, in particular, are vital for their antioxidant properties. They can donate hydrogen atoms and react with free radicals, neutralizing them and preventing oxidative damage to cells. Furthermore, these phenolic hydroxyl groups can form hydrogen bonds with biomolecules such as proteins and enzymes, influencing their activity. The ketone functional groups present in baicalein have the ability to undergo reactions with reducing agents found in living organisms, such as glutathione. This interaction serves to further augment the antioxidant properties of baicalein. The keto group in baicalein enhances its lipophilicity, hence facilitating its permeation and bioavailability across cell membranes. The binding of glucose molecules to baicalein occurs via glycosidic linkages, which may influence the bioactivity of BA. The formation of glycosidic linkages may enhance the water solubility of BA, facilitating its absorption by organisms. Alternatively, the formation of glycosidic linkages might alter the molecular polarity of BA, hence influencing its interaction with biological targets (Wang et al., 2024; Wang et al., 2022; Hu et al., 2022). The molecular dynamics simulation technique used in this study can handle the complex dynamic processes of biological macromolecules, providing a unique advantage in studying protein-ligand interaction relationships. By utilizing various computer-aided drug design methods, the study uncovers mechanisms relevant to disease impact and validates them *in vitro*, offering new ideas and directions for drug discovery and disease treatment.

## 2 Materials and methods

### 2.1 Cell lines

Mouse mammary cancer cell line 4T1, normal human breast epithelial cell line MCF-10A and BC cell lines (MCF-7 and MDA-MB-231) were purchased from the National Cell Collection Centre and maintained in the laboratory of the Department of Cell Biology at Jilin University (Jilin, China).

All cell lines were cultured in high-glucose DMEM containing 10% fetal bovine serum and 1% streptomycin-penicillin. All cells were incubated at 37°C with 5% CO_2_ for 2–3 days for passaging. Cells in the logarithmic growth phase with optimal growth conditions were used for the next steps of the experiment.

### 2.2 Drug and reagents

Chinese medicine monomer Baicalin (Must Bio-Technology, China), Cell culture medium DMEM (Gibico; Montana, United States), special medium for MCF-10A (Novozymes, Tianjin China), fetal bovine serum (CLARK, Richmond, United States), trypsin (Solembo, China), CCK8 kit (Investgent, United States), rabbit polyclonal antibody to GAPDH(Servicebio, GB15004-100, United States), rabbit monoclonal antibody to TNF-α (Servicebio, GB11188 100, United States), rabbit polyclonal antibody to JAK2(Proteintech, 17670-1-AP, China), rabbit polyclonal antibody MMP9(Wanleibio, WL03096, China), horseradish peroxidase labelled sheep anti-rabbit IgG secondary antibody (Proteintech, China). Multi-function enzyme labeler (Thermo, Vari-oskan LUX), exposure machine (Bio-Rad, YSTEM GelD), incubator (Biobase, QP-160).

### 2.3 CCK-8 assay

Select MDA-MB-231, 4T1, MCF-7, and MCF-10A cells in the logarithmic growth phase, digest them with trypsin, and count them after resuspension. Inoculate 1 × 10^4^ cells per well in 96-well plates. After 24 h to allow the cells to adhere, divide the cells into the blank group, control group, and drug-additive group. The blank group contains only medium without cells, the control group contains cells and medium without the drug, and the drug-additive group contains cells, medium, and varying concentrations of the drug.

The drug-additive group was treated with BA at concentrations of 2.5, 5, 10, 20, and 40 μM, with three parallel wells for each concentration. After 24, 48, and 72 h of incubation, the original medium was aspirated from each well. The CCK-8 reagent was prepared in a 1:10 ratio with DMEM, 100 µL was added to each well, and the samples were incubated at 37°C for 30 min. The OD value at 450 nm was measured using an enzyme marker to assess the effect of BA on the growth and proliferation of MDA-MB-231, 4T1, MCF-7, and MCF-10A cells.

Viability was calculated using the formula (%) = [(OD dosing group - OD blank group)/ [OD control group - OD blank group] × 100%, calculated and plotted using GraphPad Prism10 software.

### 2.4 Clonogenic assay

MDA-MB-231 and 4T1 cells in the logarithmic growth phase were digested with trypsin and counted. These cells were resuspended in a drug-containing medium with BA at concentrations of 0, 10, and 20 µM to create cell suspensions. The suspensions were then inoculated into 6-well culture plates at a density of 400 cells per well. The medium was changed every 3 days. The cultures were maintained until the number of cells exceeded 50 on the 14th day or until most single clones had more than 50 cells.

After the clone formation experiment, the clones were washed once with PBS. Each well was then treated with 1 mL of 4% tissue fixative for 30–60 min (min), followed by another PBS wash. Next, 1 mL of crystal violet staining solution was added to each well, and the cells were stained for 10–20 min. The wells were washed several times with PBS and air-dried, and photographs were taken of the entire six-well plate as well as each individual well.

### 2.5 Network pharmacological prediction of BA mechanism of action

#### 2.5.1 Acquisition of BA-related targets

Three compound target prediction platforms were utilized to identify baicalin-related targets: Swiss Target Prediction (http://www.swisstargetprediction.ch), SEA (https://sea.bkslab.org) and Pharmmapper (https://lilabecust.cn/pharmmapper/index.html). Targets with a Probability greater than 0 in the Swiss Target Prediction platform, targets specific to human species in the SEA platform, and targets with a z-score greater than 0 in the Pharmmapper platform were selected for further network analysis.

#### 2.5.2 TNBC related target acquisition

TNBC-related targets were identified using the Genecards (https://www.genecards.org) and OMIM (https://omim.org) databases. Targets with a Relevance score greater than 10 in the Genecards database, as well as all targets from the OMIM database, were selected for further network analysis.

#### 2.5.3 Acquisition of intersecting targets of BA and TNBC

The intersection of BA and TNBC-related targets was performed using Origin 2023 Academic Edition software to identify potential targets for BA therapy of TNBC.

#### 2.5.4 Construction of PPI protein interaction network and ‘BA-target-TNBC’ network

The protein interaction network was analyzed using the STRING database for the intersecting targets, with the organisms set to *Homo sapiens* and the minimum required interaction score set to 0.400 to obtain the PPI protein interaction network file. The PPI protein interaction network and ‘BA-target-TNBC’ network diagram were drawn using Cytoscape 3.9.0 software, and the core targets were identified based on the Degree value of the targets in the PPI protein interaction network.

#### 2.5.5 GO and KEGG enrichment analysis

GO and KEGG enrichment analyses of the core targets were performed using R 4.2.2 software and the Microbiology online mapping platform. The GO functional annotation and KEGG signaling pathways were obtained, and GO and KEGG bubble plots were generated based on the –Logb (Jiang et al., 2020) p-value, with values <0.05 considered statistically significant. The signaling pathways with the highest correlation were then visualized using R software.

### 2.6 Molecular dynamics simulation

Molecular dynamics simulations of the complexes of JAK2, MMP9, and TNF-α proteins with BA were performed using Gromacs2022 software. The protein force field chosen was Amber14sb, while Gaff2 was selected as the ligand force field. To add solvents to the protein-ligand system and create a water box with a periodic boundary of 1.2 nm, the SPC/E water model was used. The Particle Mesh method was employed to calculate the long-range electrostatic interactions. The Monte Carlo Ion Placement method was also used to introduce the necessary amount of sodium and chloride ions to neutralize the charge of the entire system. Before the formal simulation, system energy minimization and equilibration were conducted through the following steps: (1) Energy minimization was performed for each system using a 50,000-step steepest descent algorithm (Stop minimization when the maximum force <1,000 kJ/mol). (2) Each system was pre-equilibrated with a constant number of particles, volume, and temperature (310 K) for 50,000 steps, using a step size of 2 fs. (3) The entire system was further pre-equilibrated with a constant number of particles, pressure (1 atm), and temperature (310 K) for 50,000 steps, also with a step size of 2 fs. Following energy minimization and equilibration, molecular dynamics simulations were conducted without constraints for 100 ns with a step size of 2 fs, and structural coordinates were saved every 10 ps.

### 2.7 Western blotting

MDA-MB-231 cells during the period of rapid development were selected and exposed to concentrations of 0, 5, 10, and 20 µM BA for 24 h. The SDS-PAGE electrophoresis was performed using a 10% and 12.5% separator gel. The transfer apparatus was placed on ice to maintain a constant pressure of 100 V for 60 min to transfer the proteins to the PVDF membrane. The membrane was then blocked with milk for 60 min. Primary antibodies for GAPDH (1:4000), TNF-α (1:400), JAK2 (1:750), and MMP9 (1:1500) were incubated overnight at 4°C. The secondary antibody was incubated at room temperature for 2 h. Protein expression was detected using chemiluminescence (ECL).

### 2.8 Statistical analysis

The data were analyzed using SPSS 22.0 statistical software. The measurement data were presented as mean ± standard deviation (x ± s). One-way ANOVA was employed for comparisons when chi-square test assumptions were satisfied, with post-hoc comparisons made using the Least Significant Difference (LSD) test. When chi-square test assumptions were not met, the student’s t-test was performed. Statistical significance was considered when the *p*-value was <0.05.

## 3 Results

### 3.1 BA inhibits BC cell viability and clone-forming ability

In order to study the inhibitory effect of BA on TNBC cell proliferation, we applied CCK8 assay to calculate the effects of different concentrations of baicalin on the cell viability of 231 cells. Our results showed that the IC_50_ concentration of BA against 231 cells was 28.54 μM at 24 h, 23.05 μM at 48 h, and 17.35 μM at 72 h. Thus, we selected 2.5, 5, 10, 20, and 40 μM BA for the therapeutic effect on different breast cancer cell lines. BA’s inhibition of BC cell viability and clone formation was examined. The biological impact of BA on BC cells was assessed using the CCK8 assay to measure cell viability in human BC cell lines MDA-MB-231, MCF-7, 4T1, and mammary epithelial MCF-10A cells. Our results shown that BA significantly inhibited the viability of MDA-MB-231, MCF-7, and 4T1 cells, as demonstrated in Figures 1C–F. The effects of BA on BC cell viability were concentration-dependent. At a concentration of 20 μM, BA has a stronger inhibitory effect on tumor cell activity compared to normal mammary epithelial cells. Furthermore, BA effectively inhibits the viability of the TNBC cell line within 48 h (h) of administration. Within 48 h of dosing, the viability of the TNBC cell line was more significantly inhibited by BA compared to the MCF-7 cell line. The effects of BA on the capacity of MDA-MB-231 and 4T1 cells to form colonies were also investigated. Figures 1G,H demonstrates that BA treatment effectively decreased the colony-forming capacity of TNBC cells in concentration-dependent manner. The number of clones generated in the group treated with 20 µM BA was considerably lower compared to the control group. The results suggest that BA prevents the survival and ability of BC cells to form clones, and its effects are influenced by time and concentration. BA shows therapeutic properties against TNBC at the cellular level, though the exact mechanism remains unclear. Therefore, a network pharmacology approach was employed to predict its potential targets and mechanisms of action.

### 3.2 Key targets for pharmacological prediction in the BA-TNBC network

#### 3.2.1 Acquisition of related and intersection targets

**FIGURE 2:**
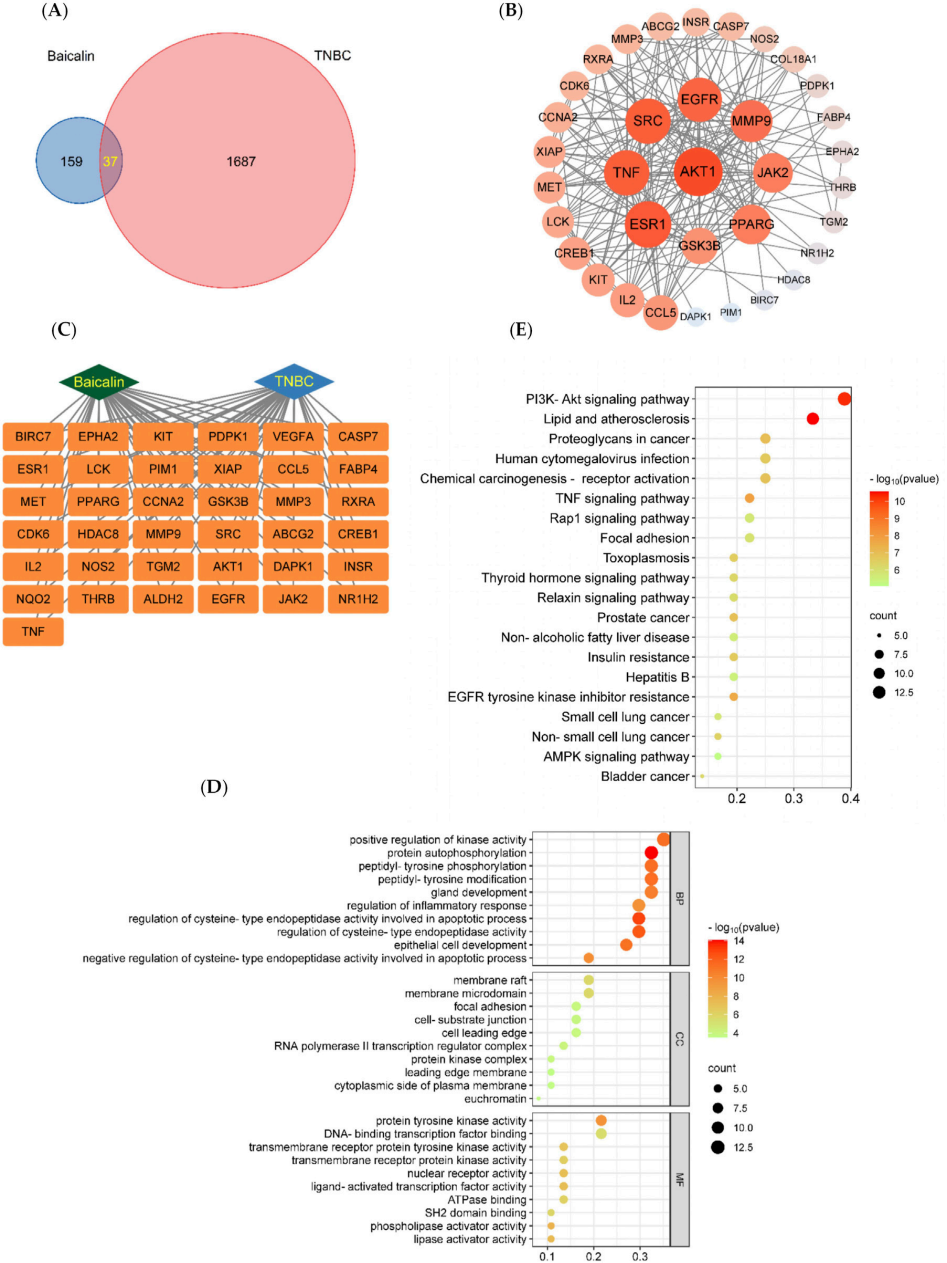
BA network pharmacology analysis. **(A)** The targets of BA intersect with the targets of TNBC. **(B)** Protein-protein interaction (PPI) network of baicalin. The node represents the protein, and the edge represents the correlation between the proteins. The size and color depth of the nodes are positively correlated with their degrees, the larger the node is, the greater the corresponding degree is, and the greater the corresponding degree of red is. **(C)** The “BA-Target-TNBC” network, **(D)** BA-TNBC GO Bubble Chart, Bubble diagram of biological processes (BP). Bubble diagram of cellular components (CC). Bubble diagram of molecular functions (MF). The Y-axis and X-axis of the above three plots indicate the full names of processes and gene ratios, respectively. The color and size of each bubble are based on P-value and gene counts, respectively. **(E)** BA-TNBC KEGG Bubble Chart. Node size and color are proportional to the size of the degree value.

A total of 14, 35, and 156 BA-related targets were acquired from Swiss Target Prediction, SEA, and Phar-mapper, respectively. After merging and de-weighting, a total of 196 BA-related targets were obtained. A total of 1,647 targets associated with TNBC were obtained from the GeneCards database and 168 from the OMIM database. After merging and de-weighting, 1724 relevant targets were identified. Using Origin software, a total of 37 possible BA targets for TNBC were identified. Figure 2A displays the intersecting Wayne diagram of these targets.

#### 3.2.2 PPI protein interaction network and ‘BA-target-TNBC’ construction

The PPI protein interaction network and the ‘Chinese medicine-component-target-disease’ network were constructed using the String protein interaction analysis platform and Cytoscape software, as shown in Figures 2B,C. Nine core targets of BA were identified for the treatment of TNBC, including AKT1, ESR1, TNF, SRC, EGFR, MMP9, JAK2, PPARG, and GSK3B, and the values of the core targets are shown in Table 1.

**TABLE 1:** Core targets and degree values.

Core targets	Degree
AKT1	27
ESR1	25
TNF	24
SRC	24
EGFR	23
MMP9	21
JAK2	19
PPARG	19
GSK3B	16

#### 3.2.3 GO and KEGG enrichment analysis

GO analysis identified a total of 2,526 biological processes (BP), 105 cellular components (CC), and 250 molecular functions (MF) associated with the intersecting targets. The top ten entries from BP, CC, and MF were selected based on the -Log (10) *p*-value to create the GO bubble plots shown in Figure 2D. The BP entries were related to positive regulation of kinase activity, autophosphorylation, and more; CC entries were related to membrane valves, membrane microstructure, and similar components; MF entries were related to protein tyrosine kinase activity, transcription factor binding, and others. KEGG analysis enriched 128 signaling pathways associated with the intersecting targets, and the top 20 pathways were selected based on the -Log (10) *p*-value to create KEGG bubble diagrams shown in Figure 2E. The results indicated that the most significantly correlated signaling pathway was the PI3K/AKT signaling pathway.

### 3.3 BA-TNBC molecular docking assay

A binding energy of less than 0 kcal/mol indicates that the receptor and ligand can bind spontaneously without external energy. A binding energy below −5 kcal/mol signifies efficient binding, while a binding energy below −7.2 kcal/mol denotes strong binding. The docking binding energies of BA with the nine core targets are listed in Table 2. All binding energies are below −7.2 kcal/mol, indicating strong binding. Significantly, JAK2, MMP9, and TNF-α showed the strongest binding to BA, with binding energies lower than −10 kcal/mol. This suggests that JAK2, MMP9, and TNF-α are the three most critical targets for BA in the treatment of TNBC.

**TABLE 2:** Combined energy data.

Protein	Ligand	Binding energy
AKT1	Baicalin	−9.2
EGFR		−9.5
ESR1		−7.6
GSK3B		−9
JAK2		−10.6
MMP9		−10.9
PPARG		−9.4
SRC		−9
TNF-α		−10.9

**FIGURE 3:**
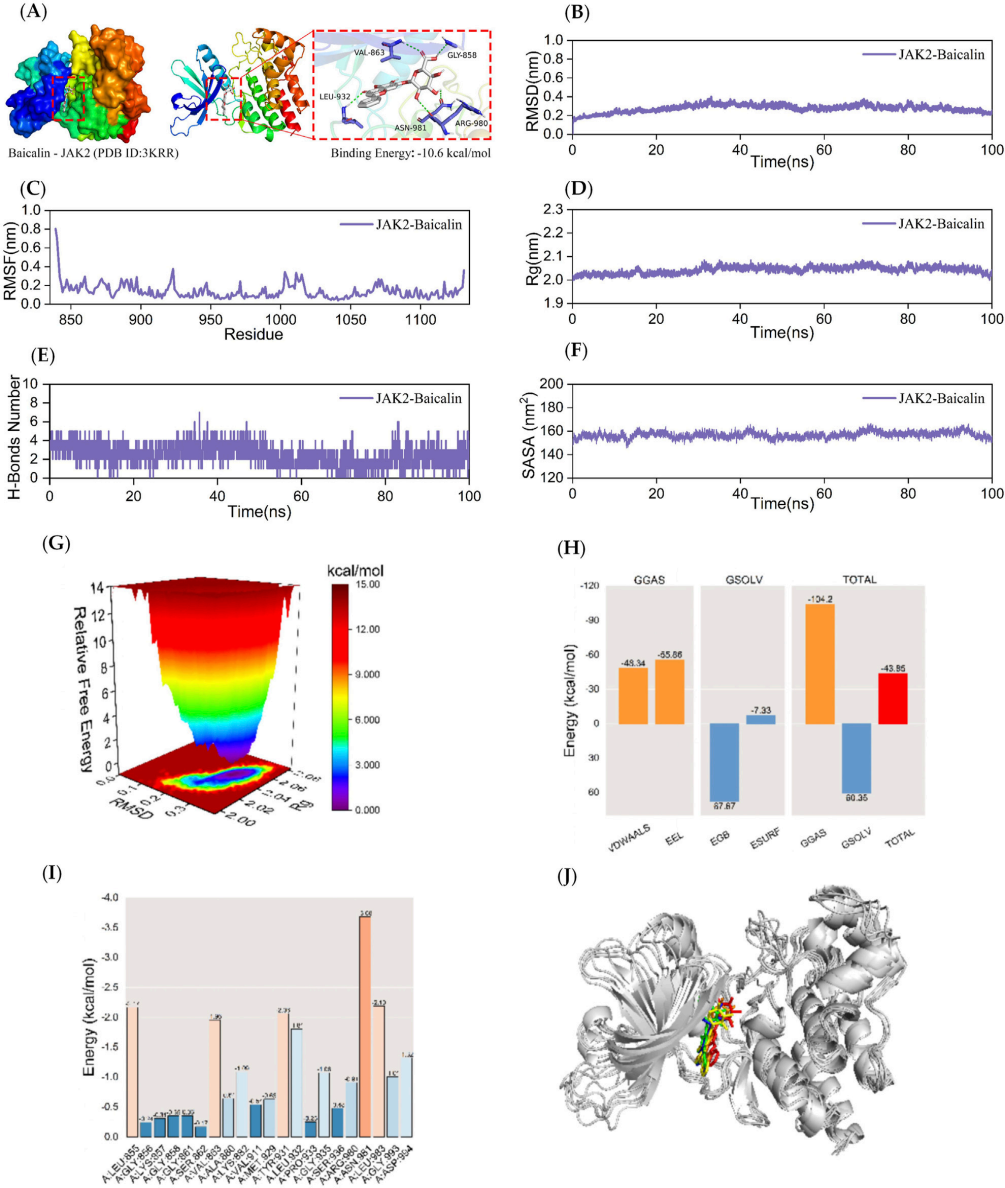
Kinetic simulations of JAK2-BA molecular docking molecules with their complexes. The figure provides a comprehensive overview of the JAK2-BA complex. Panel **(A)** visualizes the docking and binding interactions, with the protein structure depicted in color. The BA is shown as small white molecules, while small purple molecules represent the amino acid residues in the protein that interact with the ligand. Green dashed lines indicate hydrogen-bonding interactions. Panel **(B)** presents the RMSD curves for the JAK2-BA complex, while Panel **(C)** shows the RMSF curve. Panel **(D)** depicts the Rg curve of the complex, and Panel **(E)** illustrates the fluctuation curves for the number of hydrogen bonds formed between JAK2 and BA. The SASA curves are shown in Panel **(F)**. Panel **(G)** details the free energy distribution, and Panel **(H)** outlines the average binding free energy of JAK2-BA, including components such as van der Waals forces (VDWAALS), electrostatic energy (EEL), polar solvation energy (EGB), non-polar solvation energy (ESURF), molecular mechanics term (GGAS), solvation energy term (GSOLV), and the total average binding free energy. Panel **(I)** highlights the energy contributions of amino acid residues involved in the binding of BA to JAK2. Finally, Panel **(J)** compares the structural plots of the JAK2-BA complex at five-time points during molecular dynamics simulations: 0, 25, 50, 75, and 100 ns.

**FIGURE 4:**
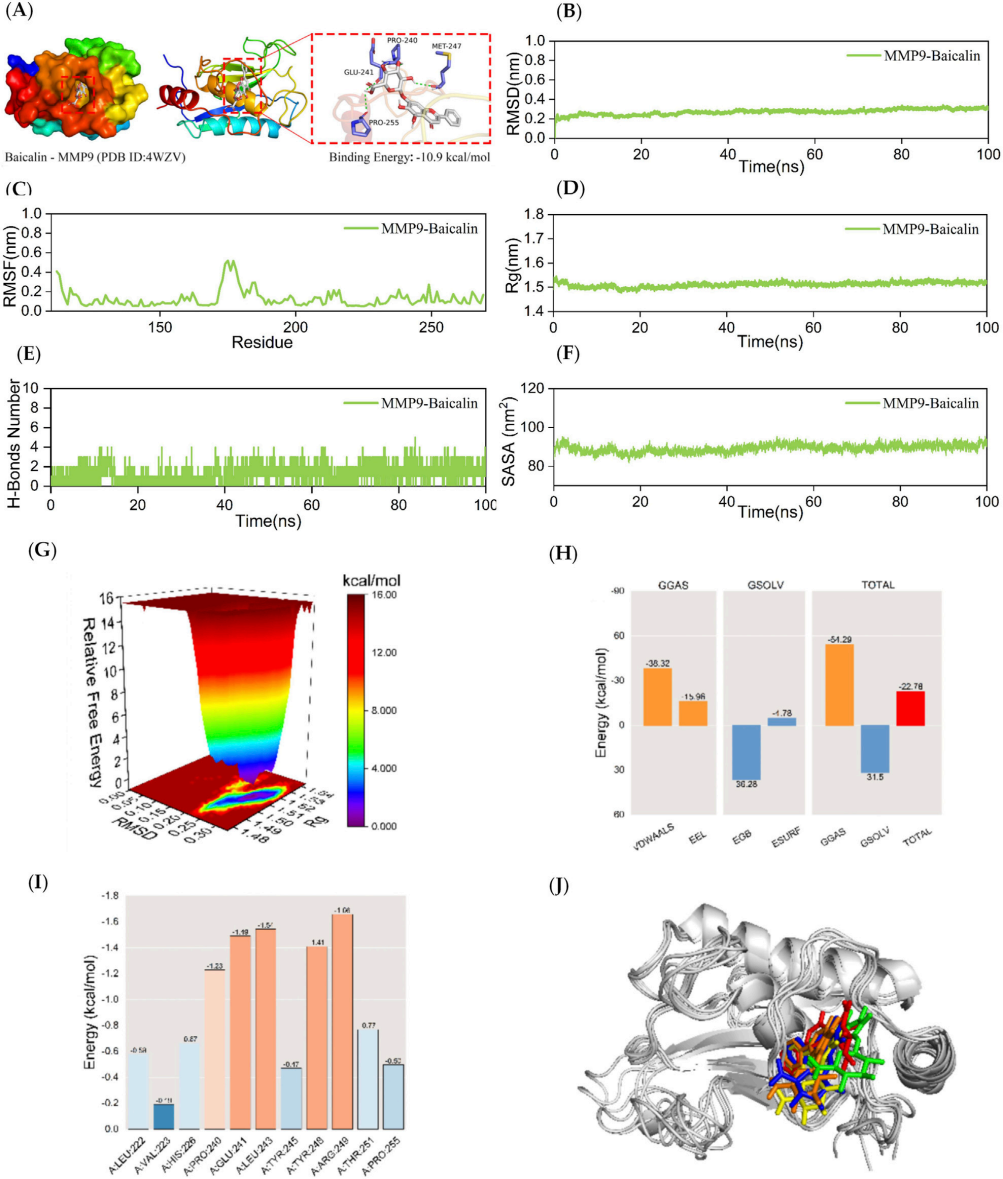
Kinetic simulations of MMP9-BA molecular docking molecules with their complexes. The figure provides a comprehensive analysis of the MMP9-BA complex. Panel **(A)** visualizes the docking and binding interactions between MMP9 and BA, where the protein structure is shown in color, small white molecules represent BA, and small purple molecules indicate the amino acid residues in the protein that interact with the ligand. The green dashed lines represent hydrogen-bonding interactions. Panel **(B)** presents the RMSD curves for the MMP9-BA complex, while Panel **(C)** shows the RMSF curve. Panel **(D)** depicts the Rg curve of the complex, and Panel **(E)** illustrates the fluctuation curves for the number of hydrogen bonds formed between MMP9 and BA. The SASA curves are presented in Panel **(F)**, and the free energy distribution is shown in Panel **(G)**. Panel **(H)** details the average binding free energy of MMP9-BA, including components such as van der Waals forces (VDWAALS), electrostatic energy (EEL), polar solvation energy (EGB), non-polar solvation energy (ESURF), molecular mechanics term (GGAS), solvation energy term (GSOLV), and the total average binding free energy. Panel **(I)** highlights the energy contributions of amino acid residues involved in the binding of BA to MMP9. Finally, Panel **(J)** compares the structural plots of the MMP9-BA complex at five different time points during molecular dynamics simulations: 0, 25, 50, 75, and 100 ns.

**FIGURE 5:**
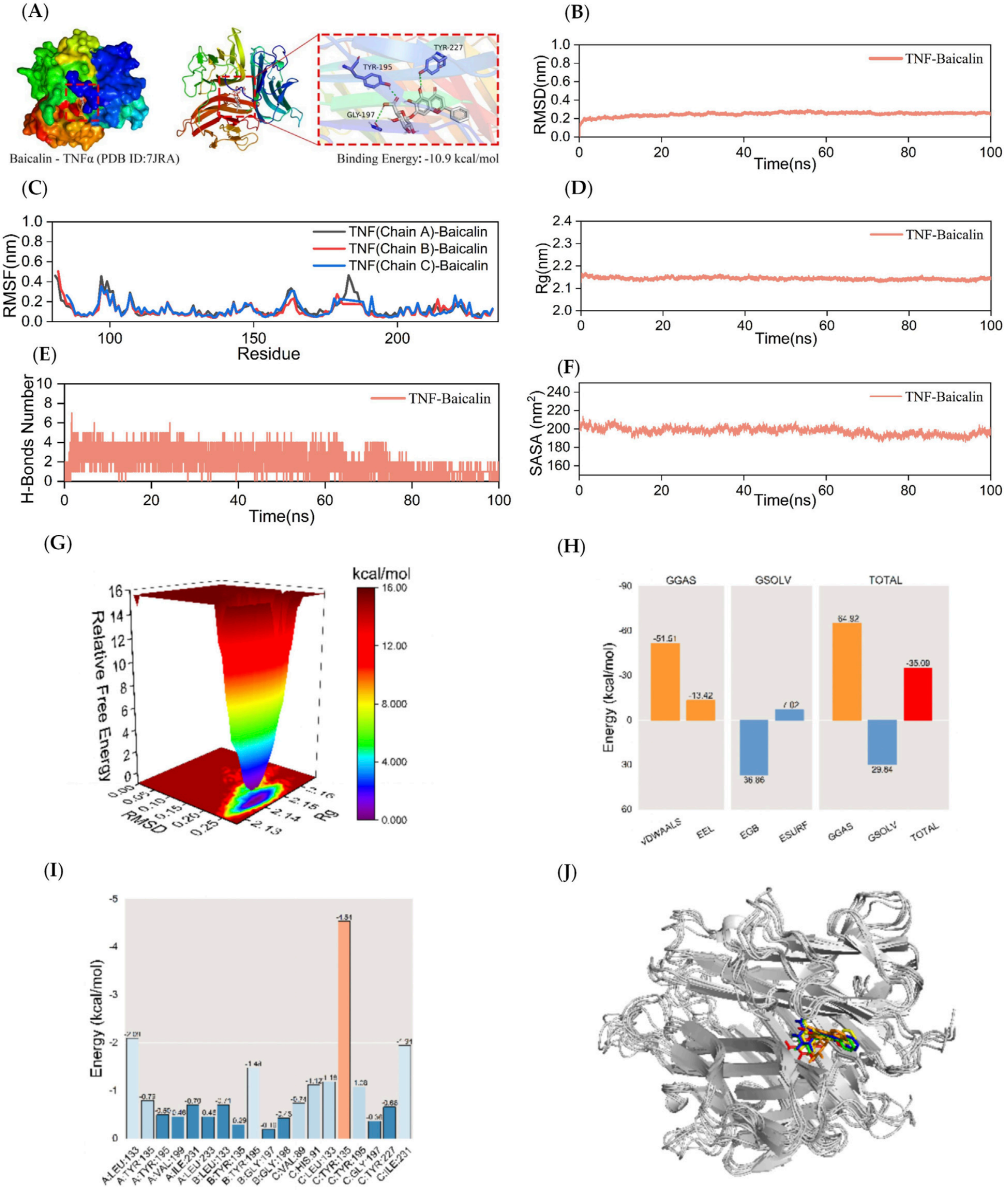
Kinetic simulations of TNF-BA molecular docking molecules with their complexes. The figure represents the kinetic simulations of TNF-α-BA molecular docking and their complexes. Panel **(A)** shows the visualization of the docking and binding interaction of TNF-α-BA molecules. The protein structure is depicted in various colors, with small white molecules representing BA, small purple molecules indicating amino acid residues in the protein that interact with the ligand, and green dashed lines representing hydrogen-bonding interactions. Panel **(B)** represents the RMSD curves of the TNF-α-BA complexes, while Panel **(C)** shows the RMSF curve of the TNF-α-BA complex. The Rg curve of the TNF-α-BA complex is displayed in Panel **(D)**, and Panel **(E)** illustrates the fluctuation curves of the number of hydrogen bonds formed between TNF-α and BA. The SASA curves of the TNF-α-BA complexes are presented in Panel **(F)**, and the free energy distribution of the TNF-α-BA complexes is shown in Panel **(G)**. Panel **(H)** details the average binding free energy of TNF-α-BA, including components such as VDWAALS (van der Waals force), EEL (electrostatic energy), EGB (polar solvation energy), ESURF (non-polar solvation energy), GGAS (molecular mechanics term), GSOLV (solvation energy term), and TOTAL (average binding free energy). Panel **(I)** highlights the energy contributions of amino acid residues involved in BA binding to TNF-α. Lastly, Panel **(J)** provides comparative structural plots of the TNF-α-BA complex at five moments during molecular dynamics simulations: 0, 25, 50, 75, and 100 ns.

The visualization of the docking data of MMP9 and TNF-α with BA was performed using PyMOL 2.3.0 software. In Figure 3A, it can be observed that BA formed five hydrogen bond interactions with amino acid residues GLY-858, VAL-863, LEU-932, ARG-980, and ASN-981 in the JAK2 protein. Similarly, in Figure 4A, BA formed four hydrogen-bonding interactions. Furthermore, in Figure 5A, BA formed three hydrogen-bonding interactions with amino acid residues TYR-195, GLY-197, and TYR-227 in the TNF-α protein. The docking results revealed that BA formed multiple hydrogen bond interactions with JAK2, MMP9, and TNF-α proteins. The binding energies for all three interactions were below −7.2 kcal/mol-1, indicating a strong binding effect. This suggests that BA is likely to affect the structural function and bioactivity of these proteins, making it a potential core component for the treatment of TNBC.

Since semi-flexible docking used for molecular docking cannot account for the flexibility of the protein structure, temperature, pressure, solvent effects, etc., further analysis is required. To demonstrate the extent and stability of the binding between the compounds and the proteins, this study performs 100 ns molecular dynamics simulations of the respective complexes of JAK2, MMP9, and TNF-α with BA.

### 3.4 Stability and gibbs free energy analysis of JAK2-BA complexes

MD simulations of JAK2-BA complexes were conducted for 100 ns to examine the dynamic characteristics acquired from molecular docking. The following parameters were measured in the molecular dynamics simulation trajectory: root mean square deviation (RMSD), root mean square fluctuation (RMSF), radius of gyration (Rg), number of hydrogen bonds between the protein and ligand in the complex, solvent-accessible surface area (SASA), and relative free energy distributions. The MM/GBSA method was also used to calculate the average binding free energies between JAK2 and BA, as well as the energy contributions of amino acid residues between JAK2 and BA. The structures of the complexes at five time points (0, 25, 50, 75, and 100 ns) during molecular dynamics simulations were also compared.

The RMSD curve serves as a measure of the stability of protein-ligand complexes. A lower RMSD value indicates a lesser alteration in the overall structure of the complex, indicating a higher level of stability (Neupane et al., 2024). Figure 3B demonstrates that the RMSD curve of the JAK2-BA complex achieved a condition of stability after 40 nanoseconds. The RMSD curve of the JAK2-BA complex showed a consistent fluctuation range of 1 nm throughout the whole process, without any significant fluctuations. This indicates that the stability of the complex generated by JAK2-BA, as analyzed from the perspective of RMSD, is remarkable.

The RMSF curve quantifies the extent of fluctuation in amino acid residues within proteins during kinetic simulation. A higher RMSF value indicates greater fluctuation in the amino acid residue, while a lower RMSF value suggests minimal fluctuation. Figure 3C indicates that the RMSF curves of the JAK2-BA complex show fluctuations that remain within a range of 1 nm during the 100 ns simulation, without any significant variations. The JAK2 protein’s terminal amino acid residues are located in the periphery of the protein’s overall structure, resulting in moderate variations of around 0.8 nm. The aforementioned data suggests that the complex produced by the addition of BA is stable and has no effect on the stability of amino acid residues in the JAK2 protein.

The Rg is a measure of the compactness and stability of a structure. A higher Rg suggests that the system experiences more significant expansion during the kinetic simulation, while a lower Rg indicates that the system remains compact and stable throughout the simulation. Figure 3D demonstrates that the Rg curves of the JAK2-BA complexes showed a fluctuation range of around 2 nm. These curves remained smooth and did not display significant variations. These findings suggest that JAK2 and BA formed a strong and stable complex, and the presence of BA did not significantly alter the overall structure of the protein. To analyze the changes in hydrogen bonding of the JAK2-BA complex, the hydrogen bonds produced during a 100 ns molecular simulation were examined. As shown in Figure 3E, the number of hydrogen bonds between the JAK2-BA complexes remained essentially stable at 2-4 throughout the simulation.

SASA is an important component in the investigation of protein structure folding and stability. This work aimed to examine the effect of BA on the SASA of the JAK2 protein. The SASA values of JAK2-BA complexes were evaluated and investigated to achieve this objective. The data shown in Figure 3F revealed that the SASA curve of the JAK2-BA complex remains consistently stable, with a fluctuation range of around 150 nm^2^ and no significant variations. This suggests that the complex displays a high level of stability.

The Gibbs relative free energy in the free energy landscape (FEL) was determined by calculating the RMSD value and Rg value using the Gromacs built-in script g_sham and xpm2txt.py script. The resulting FEL was then visualized by plotting the RMSD value, Rg value, and Gibbs relative free energy on the X, Y, and Z-axes. The FEL map was used to depict the conformation with the lowest energy during the whole process of simulating the structural dynamics of the complex. When the interaction between the protein and ligand is weak or unstable, the FEL map shows many rough surface clusters with minimal energy. Conversely, strong and stable interactions result in almost single and smooth energy clusters in the potential energy distribution. The dark purple/blue areas in Figure 3G indicate energy minima, which correspond to the most stable structures. On the other hand, the red/yellow spots represent unstable structures. The free energy distribution of the JAK2-BA complex shown in the picture demonstrates the presence of a single minimum energy cluster with a highly concentrated distribution of energy clusters. This suggests that the complex formed between JAK2 and BA is very stable.

After stabilizing the complex system, the MM/GBSA binding free energy of the JAK2 protein with BA was calculated. As shown in Figure 3H, the average binding free energy is −43.85 kcal/mol, indicating a strong binding affinity between the JAK2 protein and BA. This is further evidenced by Figures 4H, 5H, where BA shows the highest binding affinity to the JAK2 protein.

Figure 3I illustrates the interactions between BA and several amino acid residues in the JAK2 protein. Among these, ASN-981 showed the best binding energy lowest binding energy to BA with a value of −3.68 kcal/mol, highlighting its significant role in the binding process.

The binding stability of the complex was assessed through MD simulations by comparing the complex conformations at different time points. Figure 3G shows that BA remains bound to the same position on the JAK2 protein at 0, 25, 50, 75, and 100 ns without significant changes, indicating excellent binding stability.

### 3.5 Stability and gibbs free energy analysis of MMP9-BA complexes

Afterwards, a 100 ns MD simulation investigation on the MMP9-BA complex was conducted. Figure 4B demonstrates that the RMSD curve of the MMP9-BA complex achieved a somewhat stable state after 30 ns. The RMSD curve of the MMP9-BA complex showed a consistent fluctuation range of 1 nm throughout the whole process, without any significant variations. This suggests that the complex formed by MMP9-BA is stable when assessed based on RMSD. Figure 4C indicates that the RMSF curves of the MMP9-BA complexes fluctuated within a range of 0.6 nm during the entire 100 ns simulation. This suggests that the presence of BA has a minor effect on the stability of the amino acid residues in the MMP9 protein. Therefore, the formed complexes are considered stable. As shown in Figure 4D, the Rg curves for the MMP9-BA complexes showed fluctuations within a range of approximately 1.5 nm, with a smooth process and minimal large deviations. This suggests that MMP9 forms a tight and stable complex with BA, and the addition of BA does not significantly alter the overall structure of the MMP9 protein. Figure 4E shows that the number of hydrogen bonds in the MMP9-BA complexes remained stable between 1 and 3 throughout the simulation. The SASA values for the MMP9-BA complex were also analyzed, as depicted in Figure 4F. The results demonstrated that the SASA curve fluctuated within a range of about 90 nm^2^ and remained stable without significant variations, indicating high stability of the complex. Furthermore, the free energy distribution diagram of the MMP9-BA complex revealed two relatively small and concentrated energy clusters, suggesting that the complex between MMP9 and BA displayed excellent stability. After stabilizing the complex system, the MM/GBSA binding free energy of the MMP9 protein with BA was calculated. Figure 4H showed that the average binding free energy of −22.78 kcal/mol indicated a strong binding affinity between MMP9 and BA. Figure 4I revealed that BA interacts with several amino acid residues in the MMP9 protein, with ARG-249, LEU-243, and GLU-241 showing binding energies of −1.66, −1.54, and −1.49 kcal/mol, respectively. This suggests that ARG-249, LEU-243, and GLU-241 are crucial for the binding of BA to MMP9. Figure 4G demonstrates that BA and MMP9 maintain their binding position consistently at the five-time points (0, 25, 50, 75, and 100 ns) without significant changes, indicating excellent binding stability of the complex.

### 3.6 Stability and gibbs free energy analysis of TNF-α-BA complexes

A 100 ns MD simulation analysis for the TNF-α-BA complex was conducted. The RMSD curve for the TNF-α-BA complex reached a relatively stable state after 20 ns, with fluctuations within 0.4 nm throughout the simulation, indicating a stable complex (Figure 5B). The RMSF curves for the TNF-α-BA complex fluctuated within 0.6 nm over the 100 ns period, suggesting that the addition of BA increases the flexibility of the amino acid residues in the TNF-α protein compared to the JAK2 and MMP9 proteins (Figure 5C). The Rg curves for the TNF-α-BA complex showed a fluctuation range of approximately 2.2 nm, with smooth progression and no significant deviations, indicating that TNF-α forms a tight and stable complex with BA, and the addition of BA does not significantly alter the overall structure of the TNF-α protein (Figure 5D). As shown in Figure 5E, the number of hydrogen bonds between TNF-α-BA complexes remained stable at 1-4 throughout the simulation. The SASA values of the TNF-α-BA complexes were analyzed, and the results (Figure 5F) indicate that the SASA curve remained stable with a fluctuation range around 200 nm^2^, suggesting high stability of the complex. The free energy distribution diagram of the TNF-α-BA complex reveals a single small energy cluster with concentrated distribution, indicating good stability of the complex (Figure 5G). After stabilizing the complex system, the MM/GBSA binding free energy of the TNF-α protein with BA was calculated. The average binding free energy was −35.09 kcal/mol, indicating a strong binding affinity between TNF-α and BA (Figure 5H). BA forms interactions with several amino acid residues in the TNF-α protein, with TYR-135 in the C chain showing the best binding energy of −4.54 kcal/mol, suggesting its significant role in the binding of BA to TNF-α (Figure 5I). Figure 5G shows that BA binds to the same position on the TNF-α protein at five different time points (0, 25, 50, 75, and 100 ns) without significant changes, indicating good binding stability.

### 3.7 Effect of BA on the expression of JAK2, MMP9, and TNF-α in MDA-MB-231 cell line

**FIGURE 6:**
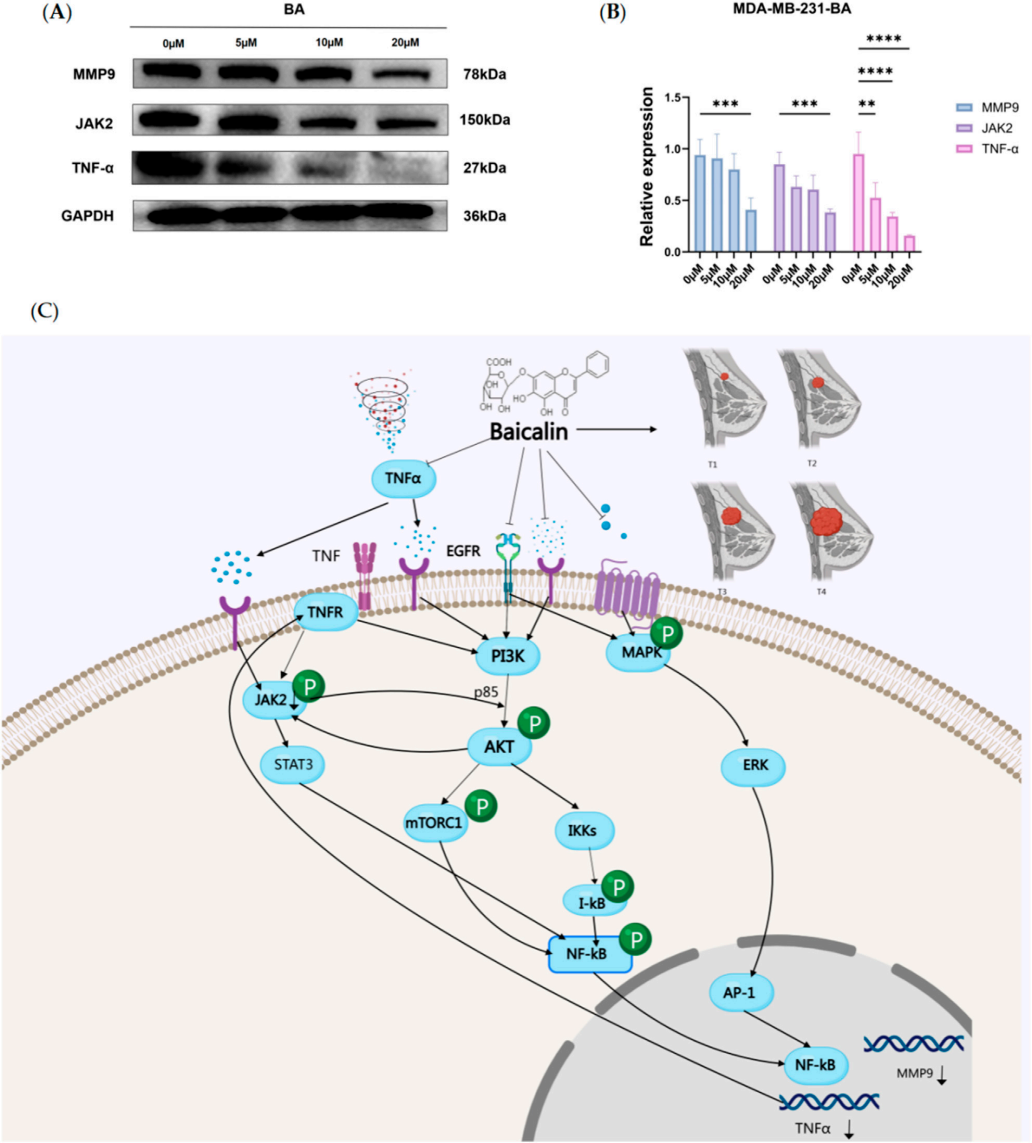
**(A, B)** Effect of BA on JAK2, MMP9, and TNF-α protein expression in MDA-MB-231 cell. The figure shows the protein expression analysis of JAK2, MMP9, and TNF-α in MDA-MB-231 cells after treatment with different concentrations of BA for 24 h. *p < 0.05; **p < 0.01; ***p < 0.001. **(C)** Proposed signaling network of Baicalin’s action in TNBC, highlighting the regulation of MMP9, TNFα, and JAK2 pathways.

Through bioinformatics analysis, we screened out the core targets of BA on TNBC. To further explore the mechanisms of BA on these core targets, we assessed the expression levels of JAK2, MMP9, and TNF-α proteins in the MDA-MB-231 cell line following treatment with various concentrations of BA for 24 h. The results indicated that, compared to the control group, the protein expression levels of JAK2 and MMP9 were significantly reduced at a BA concentration of 20 μM, while the expression level of TNF-α decreased significantly with increasing BA concentrations (p < 0.001). (Figure 6A,B).

Based on the previous results, we understand that BA regulates the development of TNBC through multiple targets and pathways, primarily through PI3K/Akt signaling pathway, which plays a crucial role in cell survival, proliferation, and immune regulation. Multiple pathways involved affect the expression of target proteins. This study mainly focuses on exploring the specific binding conformation between baicalin and target proteins. Therefore, we evaluated the expression of target proteins in TNBC cells under different concentrations of baicalin. Based on network pharmacology results and existing literature, we predicted the potential mechanism of BA in treating TNBC through target protein expression levels.

Literature has shown that PI3K/Akt is overactivated in TNBC (Costa et al., 2018; Zhang HP. et al., 2024; Sirek et al., 2024), and after PI3K/Akt activation, it works synergistically with the MAPK pathway to regulate MMP9 expression through NF-κB. NF-κB, as a transcription factor, binds to cis-regulatory elements on the MMP9 promoter to regulate MMP9 transcription (Mao et al., 2024). JAK2 and PI3K/Akt have bidirectional regulation, where growth factors or cytokines activate JAK2, and Akt can directly phosphorylate JAK2 to activate it. JAK2 activation can in turn activate the PI3K/Akt signaling pathway, forming a positive feedback loop (Jin et al., 2008; Luo et al., 2023; Koopmans et al., 2014). TNFα is activated by PI3K/Akt through NF-κB, while TNFα itself can promote PI3K/Akt activity, forming a positive feedback loop (Yang et al., 2015; Gajate et al., 2017). In TNBC, the interactions between molecules or co-regulatory networks, such as NF-κB may simultaneously participate in the regulation of MMP9 and TNFα, while JAK2 may form cross-pathways with PI3K/Akt, jointly affecting tumor progression. These mechanisms work together to influence tumor cell survival, proliferation, and inflammatory responses.

As shown in Figure 6, BA enters cells and inhibits PI3K/Akt signaling pathway to activate transcription factor NF-κB, suppressing activated Akt through IKK phosphorylation, thereby inhibiting NF-κB nuclear translocation and binding to MMP9 promoter region. It simultaneously inhibits MAPK pathway activation of AP-1 transcription factor, further reducing MMP9 transcription. JAK2, as a non-receptor tyrosine kinase, can be activated by cytokines like TNFα. When TNFα binds to BA, it may inhibit JAK2 activation. Meanwhile, the inhibited PI3K/Akt signaling pathway can also reduce JAK2 activation. After PI3K/Akt signaling pathway inhibition, TNFα transcription is directly downregulated by reducing IKK/NF-κB pathway activation. In the tumor microenvironment, TNFα binding to BA can itself inhibit PI3K/Akt pathway through TNFR receptor, forming an autocrine loop. Reduced Akt activation and further phosphorylation can decrease mTORC1 activation, thereby reducing tumor cell proliferation, survival, and metabolism, forming a positive cycle.

The PI3K/Akt pathway regulates MMP9, JAK2, and TNFα through multiple dimensions in TNBC, becoming a key therapeutic target. It is recommended to include surface plasmon resonance (SPR) to determine the binding kinetics between baicalin and target proteins, Phos-tag gel electrophoresis to verify phosphorylation inhibition effects, and intermolecular FRET experiments to detect TNFα trimer stability in future experiments to further validate this hypothesis.

## 4 Discussion

Progress has been made in the treatment of TNBC, but the disease remains challenging due to its highly heterogeneous and rapidly progressive nature (Santuario-Facio et al., 2017). Modern medicine continues to explore new molecular targets and therapeutic strategies to improve outcomes for TNBC patients (Ganesan et al., 2024; Wu S. et al., 2024; Zhang C. et al., 2024; Chu et al., 2025; Ayvaz and Bolat, 2025; Chien et al., 2025; Dong et al., 2025). In recent years, traditional Chinese medicine (TCM) has also become a part of comprehensive TNBC treatment (Qiu et al., 2024; Meng et al., 2017; Dou et al., 2024). TCM exerts anti-tumor effects by regulating the immune system and inhibiting tumor cell proliferation, offering an additional therapeutic option for TNBC patients. However, the diversity of active ingredients in TCM and the complexity of its mechanisms lead to an unclear understanding of its specific mechanisms of action and core targets (Fakhri et al., 2024; Ru et al., 2014; Lv et al., 2024; Wu W. et al., 2024). Advances in drug extraction technology have prompted extensive research on the active monomer components of natural medicines, both domestically and internationally.

The Chinese medicine Scutellaria baicalensis, a traditional remedy for breast diseases, contains BA as one of its main active components. Research indicates that BA affects BC cells through multiple pathways. For instance, BA markedly inhibits cell migration and invasion by suppressing the activities of matrix metalloproteinases MMP2 and MMP-9, which are crucial for BC cell invasion (Zeng et al., 2020; Wu et al., 2023; Wang et al., 2013). Furthermore, BA reduces the proliferation, migration, and invasiveness of MDA-MB-231 cells by downregulating tissue-specific nuclear machinery binding protein 1 (SATB1) (Ma et al., 2016; Lee et al., 2016; Chen et al., 2022). Studies have also demonstrated that BA induces apoptosis, actively promoting cancer cell death and thereby inhibiting tumor growth, potentially through its modulation of miRNA-mediated inflammatory responses (Huang et al., 2018; Wen et al., 2024; Yuan et al., 2023; Li et al., 2022; Xu et al., 2019; Duan et al., 2019; Zhu et al., 2018). Furthermore, BA has been shown to reverse epithelial-mesenchymal transition (EMT) by targeting the β-linker protein signaling pathway, which helps to inhibit the metastasis of highly invasive BC cells (Ma et al., 2016; Zhou et al., 2017; Wang et al., 2017). These researches have demonstrated that BA has a therapeutic effect on BC, further verifying the reliability of our predicted targets.

Bioinformatics, computer-assisted drug design, network pharmacology, molecular docking, and molecular dynamics were used to identify key targets for TNBC treatment, including JAK2, MMP9, and TNF-α (Zhang et al., 2021; Deng et al., 2022). The investigation confirmed the presence of the JAK2 gene amplification phenomenon. In particular, JAK2 amplification at the 9p24 gene causes JAK2-specific dependency, indicating that JAK2 is overexpressed in TNBC and could be a potential therapeutic target (Burkhard et al., 2019; Balko et al., 2016). Furthermore, MMP9 expression is significantly higher in TNBC compared to benign breast lesion tissues. The study found that the MMP9 positive rate in TNBC tissues was 63.2%, compared to 32.5% in benign breast lesion tissues, suggesting that MMP9 plays a significant role in the development of TNBC and its high expression correlates with the tumor’s invasiveness and metastatic capabilities (Elbaz et al., 2015; Chien et al., 2018; Liu et al., 2020; Wang et al., 2019; Terkelsen et al., 2021). Regarding TNF-α, there is no direct evidence of its specific expression level in TNBC (Gong et al., 2019). Recent studies have demonstrated significant potential in targeting JAK2, MMP9, and TNF-α for medicinal purposes. Inhibitors JAK2 have demonstrated favorable effectiveness in various inflammatory and autoimmune conditions (Xing et al., 2014; Quintás-Cardama et al., 2011; Wu et al., 2015). The MMP9 inhibitors are expected to be used for regulating inflammatory response and tumor advancement (Kalhori and Törnquist, 2015; Muthukuru and Cutler, 2015). Meanwhile,TNF-α inhibitors have made significant advancements in treating inflammatory bowel disease and other illnesses (Campanati et al., 2019; Atiqi et al., 2020; Meijboom et al., 2021). The stable interaction between the JAK2, MMP9, and TNF-α proteins with BA also suggests that BA could serve as a target inhibitor for natural drug monomers.

The present study demonstrates that BA shows significant anti-TNBC effects. Research has demonstrated that BA has the ability to prevent the proliferation and migration of many cancer cells and trigger programmed cell death in tumor cells. Typically, traditional chemotherapeutic medications display significant cytotoxicity, resulting in a range of adverse effects including fever, nausea, and immunosuppression. However, BA has the ability to reduce these negative effects by regulating inflammatory responses and antioxidant mechanisms. Several studies have demonstrated that the concurrent administration of BA with chemotherapeutic medications can effectively augment the susceptibility of tumor cells to these drugs, decrease the quantity of drugs required, and simultaneously alleviate the adverse effects induced by the drugs. BA has a wider range of biological functions, including antibacterial, anti-inflammatory, and antioxidant properties, in addition to its anti-tumor actions. This makes it a promising candidate for the combination therapy of TNBC.

Although some of the mechanisms of action of BA have been revealed by existing studies, further studies are needed to fully understand its specific role in different cancers. In this study, network pharmacology was used to computationally predict the core targets of action of BA for treating TNBC and validate these targets at the protein expression level *in vitro*. This approach offers insight into the mechanism of BA’s action against TNBC. However, to further determine which target or all of them BA acts on, we will need to conduct further pharmacological inhibition or gene interference experiments for verification. In conclusion, BA can inhibit the protein expression of MMP9, TNF-α, and JAK2, along with their related signaling pathways, by stably binding to these proteins. Our study indicates that MMP9, TNF-α, and JAK2 are likely core targets of BA in the treatment of TNBC, making BA a promising candidate as a natural drug monomer offering a multi-targeted approach for treating TNBC.

## Data Availability

The raw data supporting the conclusions of this article will be made available by the authors, without undue reservation.
